# Statistical modeling and investigation of thermal characteristics of a new nanofluid containing cerium oxide powder

**DOI:** 10.1016/j.heliyon.2022.e11373

**Published:** 2022-11-02

**Authors:** Behrooz Ruhani, Mansour Taheri Andani, Azher M. Abed, Nima Sina, Ghassan Fadhil Smaisim, Salema K. Hadrawi, Davood Toghraie

**Affiliations:** a Solar Energy Naqsh-e Jahan Company, Chahar Bagh St, Isfahan, Iran; b Department of Material Science Engineering, Isfahan University of Technology, Isfahan, Iran; c Air Conditioning and Refrigeration Techniques Engineering Department, Al-Mustaqbal University College, Babylon 51001, Iraq; d Department of Mechanical Engineering, Najafabad Branch, Islamic Azad University, Najafabad, Iran; e Department of Mechanical Engineering, Faculty of Engineering, University of Kufa, Iraq; f Nanotechnology and Advanced Materials Research Unit (NAMRU), Faculty of Engineering, University of Kufa, Iraq; g Refrigeration and Air-conditioning Technical Engineering Department, College of Technical Engineering, The Islamic University, Najaf, Iraq; h Computer Engineering Department, Imam Reza University, Mashhad, Iran; i Department of Mechanical Engineering, Khomeinishahr Branch, Islamic Azad University, Khomeinishahr, Iran

**Keywords:** Thermal conductivity, Cerium oxide, Ethylene glycol, Nanofluid, Artificial Neural Network (ANN)

## Abstract

In this paper, the thermal conductivity (k_nf_) of cerium oxide/ethylene glycol nanofluid is extracted for different temperatures (T = 25, 30, 35, 40, 45, and 50 °C) and the volume fraction of nanoparticles (φ= 0, 0.25, 0.5, 0.75, 1, 1.5, 2 and 2.5%) and then k_nf_ is predicted by two methods including Artificial Neural Network (ANN) and fitting method. For both methods, the results have been presented and compared. The experiments showed that with increasing φ and temperature, the thermal conductivity ratio (TCR) of nanofluid increases. It was also observed that when the experiments are performed at high temperatures, the rate of increase in k_nf_ is much higher than the change in the same amount of φ change at low temperatures. An ANN with 7 neurons has a correlation coefficient very close to 1 and this proves that the outputs are compatible with experimental results. Also, it can be seen that the ANN could predict the thermal behavior of cerium oxide/ethylene glycol nanofluid more accurately.

## 1 Introduction

An Artificial Neural Network (ANN) is made up of neurons that work together to solve a problem [1, 2, [3], [4]]. Heat transfer fluids have various industrial applications. Conventional cooling fluids are inherently poor in heat due to their low thermal conductivity [5, 6]. Research and development activities to improve the thermal properties of fluids and phase change materials (PCMs) were always ongoing [[7], [8], [9], [10]]. Metallic solids and non-metallic materials have a much higher thermal conductivity than conventional cooling fluid. Hence, an innovative idea is to add solid particles to traditional cooling fluids to increase their thermal conductivity. Therefore, predicting the rheological behavior and heat transfer properties of nanofluids is a practical goal. Due to the high cost of heat transfer tests, it is not possible to repeat one test for different data, so predicting the results of one test for different amounts of untested data is an important issue. Due to the accurate results obtained from ANNs in predicting values, the use of this method in predicting the values of untested data has been considered in this article. In this method, by having a limited number of input data and their desired output, the network can be trained in such a way that for a wide range of input data, the desired output can be predicted with great accuracy [7, 8, 9, 10]. With advances in computer science and software, researchers using computational methods such as ANNs, Fuzzy logic, and genetic algorithms tried to model the k_nf_. ANNs were used by many researchers in various engineering systems to model the thermophysical properties of nanofluids.

Safaei et al. [11] investigated the effect of temperature and φ on the k_nf_ of ZnO–TiO_2_/ethylene glycol nanofluid using ANN and curve fitting. Their results showed that there is a good agreement between results so that the model of ANN can predict the k_nf_. Ramezanizadeh and Nazari [12] modeled the k_nf_ of Ag/water nanofluid using the ANN method. They concluded that the highest value of relative error is approximately 18.96%. Akhgar et al. [13] developed ANNs to predict the k_nf_ of MWCNT-TiO_2_/Water-ethylene glycol nanofluid. They found that ANNs can predict the empirical results better than the correlation formula. Toghraie et al. [14] designed an ANN to predict the viscosity of Ag/Ethylene glycol nanofluid. They concluded that the ANN can predict the viscosity of this nanofluid. Peng et al. [15] developed an ANN to predict the k_nf_ of Al_2_O_3_–Cu/ethylene glycol nanofluid. They showed that the ANNs can present the k_nf_ with good agreement empirical data. Ghazvini et al. [16] used ANNs to predict the k_nf_ of copper/water nanofluid. They found that the ANN is a good model to predict the k_nf_. Wang et al. [17] estimated the k_nf_ based on ANN models. They showed that the k_nf_ increases nonlinearly with the ratio of water to ethylene glycol and temperature. Jamei et al. [18] estimated the k_nf_ of various nanofluids using artificial intelligence techniques. They showed that the φ was the most influential factor among all model input parameters. Petrudi and Scurtu [19] modeled the k_nf_ and viscosity by changing the temperature and the φ using ANNs. Their results show that the highest k_nf_ and the lowest viscosity occur when a maximum temperature and φ are used. Petrudi and Rahmani [20] optimized the thermophysical properties of nanofluid using ANN. They showed that the highest k_nf_ occurs at maximum temperature and φ. Bakthavatchalam et al. [21] proposed an ANN method to predict the thermophysical properties of different nanofluids. They showed that with the inputs of temperature, φ, type, and size of nanomaterial, the ANN model illustrated a good consistency with the experimental data. He et al. [22] predicted the k_nf_ of ZnO–Ag/Water nanofluid using an ANN method. They found that an ANN method had a better ability in predicting the k_nf_. Zahmatkesh et al. [23] investigated entropy generation of nanofluid flow in the stagnation point by impinging on the cylinder axes with constant wall heat flux and uniform transpiration.

A review of previous research shows that providing empirical relationships and modeling with the help of ANNs is a suitable method that was considered by many researchers. These methods can replace repeated tests, which are time-consuming and costly. In this study, optimal ANN is obtained by considering the number of different neurons in the hidden layer and considering the least prediction error. Based on the research and studies of the authors, no research has been done on the k_nf_ of cerium oxide/ethylene glycol nanofluid using ANN.

## 2 Experimental results

In the present study, the hot wire method is used to measure the k_nf_ using a thermal analyzer KD2-Pro. Cerium oxide nanoparticles are suspended in φ = 0, 0.25, 0.5, 0.75, 1, 1.5, 2 and 2.5% in the ethylene glycol base fluid. The k_nf_ of nanofluids at T = 25, 30, 35, 40, 45, and 50 °C was obtained experimentally [8]. The diameter of nanoparticles of cerium oxide is 10–30 nm and their bulk density is 0.8–1.1 g/cm^3^. In this experiment, a two-step method using ultrasonic and magnetic stirrer has been used to produce the Cerium oxide - ethylene glycol nanofluids. To produce the nanofluids in different φ, the mass of nanoparticles is measured using a digital scale with an accuracy of 0.001 g. The weighted nanoparticles are poured into the base fluid and mixed with a magnetic stirrer for 5 h. The resulting mixture is then exposed to ultrasonic waves for 4, 8, and 12 h, and the suspension is completed by adding surfactant and controlling the acid at a point far from the isoelectric point (IEP). The resulting nanofluid had good stability and no sediment or sediment was observed in the period before the test.

**Figure 1:**
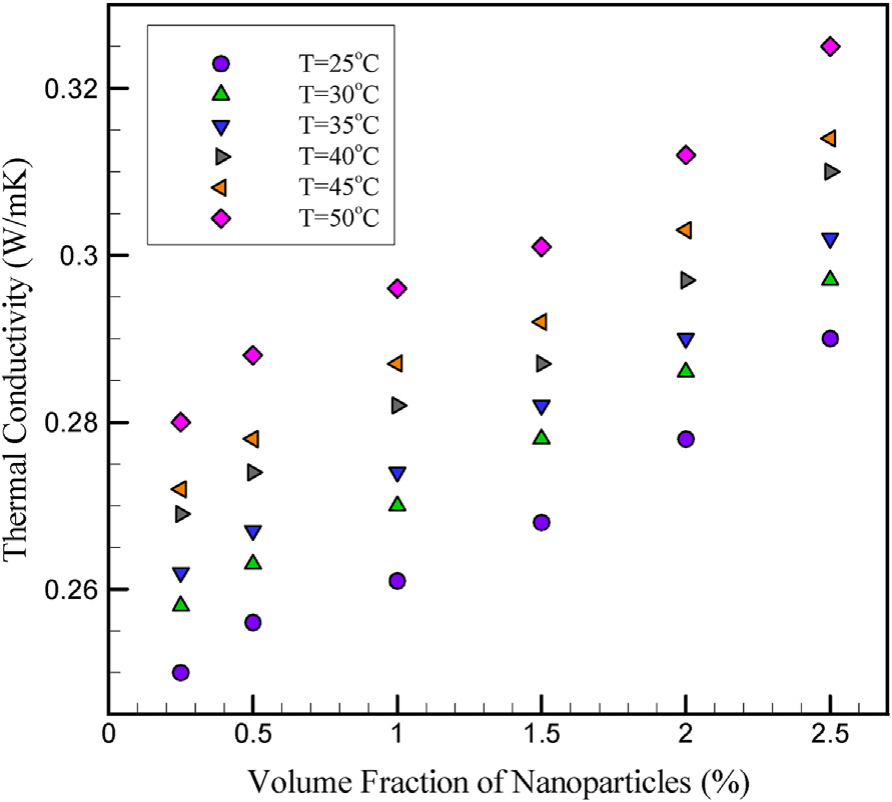
k_nf_ of cerium oxide/ethylene glycol nanofluid [8].

**Figure 2:**
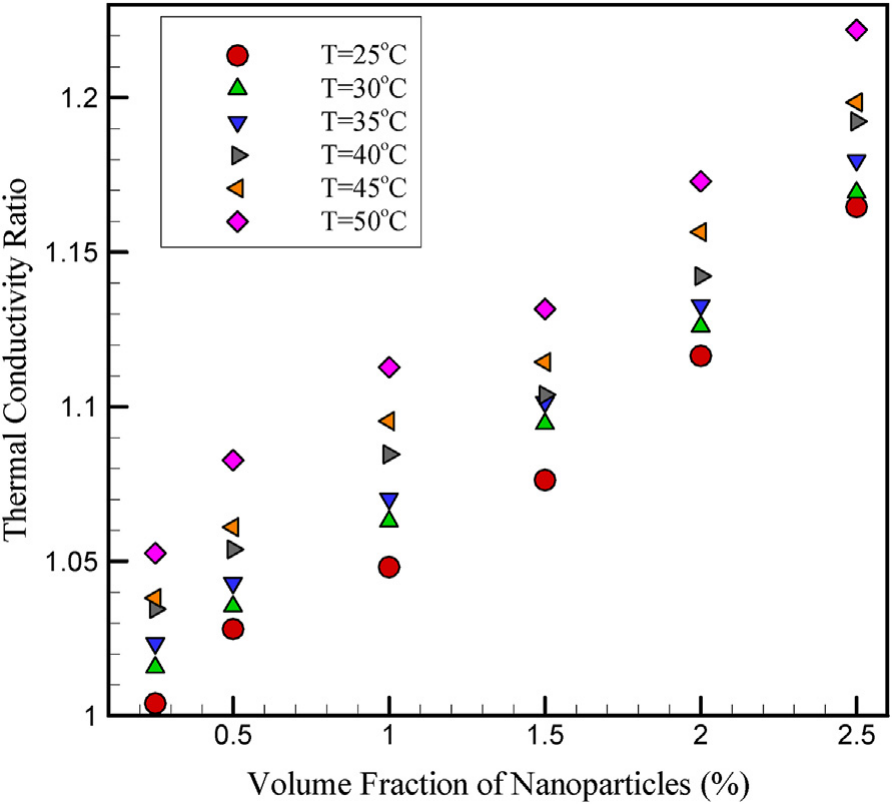
TCR of cerium oxide/ethylene glycol nanofluid [8].

Experimental data for k_nf_ and TCR of this nanofluid are shown in Figures 1 and 2. The experiments are performed in T = 25 °C–50 °C for φ = 0–2.5%. The experiments show that with increasing φ and temperature, the TCR of nanofluid increases. It is also observed that when the experiments are performed at high temperatures, the rate of increase in k_nf_ due to the change in the φ is much higher than the change in the same amount of φ change at low temperatures. Also, at higher φ, the changes in the k_nf_ of the nanofluid are much more pronounced due to the temperature change. The results show that for low φ at any constant temperature, increasing the particle concentration causes a significant increase in the k_nf_. However, at higher φ, this increase indicates a lower slope. As the φ increases and the nanoparticles come together, clusters of nanoparticles are formed. By forming these clusters, heat can travel through these solid regions faster than when passing through the fluid, thus increasing the k_nf_. In nanofluids with smaller nanoparticles, the occurrence of this phenomenon is more obvious. The joining of this phenomenon is more obvious, because in these nanofluids, at a certain φ, the distance between the nanoparticles decreases with decreasing diameter and the Van der Waals absorption forces between the particles become more intense. On the other hand, it can be concluded that no considering the effective and important parameters such as temperature, particle diameter, and size and stabilization method in the relationships of estimation models, the k_nf_ can make them inefficient and reduce their efficiency.

## 3 Artificial Neural Network

### 3.1 The neuron numbers based on the best performance

Artificial Neural Networks (ANNs) are widely used in engineering projects. If these ANNs are designed accurately, they can predict the behavior of complicated systems including linear on non-linear behavior. In the current work, the inputs are φ which consists of (φ= 0, 0.25, 0.5, 0.75, 1, 1.5, 2 and 2.5%) and temperature which includes (T = 25, 30, 35, 40, 45 and 50 °C) and the goal is predicting k_nf_ of nanofluid. The numbers of data points are 42. To predict the k_nf_, a feed-forward network is designed. This network consists of three layers: An input layer, a hidden layer, and the output layer. The output layer has only one neuron (Because only one parameter should be predicted). For the hidden layer, different neuron numbers have been tested and then the best neuron based on the performance has been selected and applied. In Table 1, the neuron numbers are sorted based on the best performance. In this table for each neuron number, the train, validation, test, and all performances are presented. It can be seen that a network with 7 neurons in the hidden layer has the best performance.

**Table 1:** The neuron numbers based on the best performance.

Neuron number	All Performance	Train Performance	Validation Performance	Test Performance
7	1.5776E-06	2.35806E-07	4.98376E-06	4.88167E-06
8	3.10805E-06	8.28894E-07	6.36938E-06	6.57152E-06
9	3.22086E-06	4.04681E-07	1.07018E-05	1.07403E-05
10	3.2997E-06	4.95204E-07	1.27729E-05	8.62755E-06
6	4.29834E-06	1.08119E-06	4.78069E-06	1.27055E-05
12	5.19269E-06	6.62598E-07	1.72185E-05	1.71901E-05
13	5.58251E-06	7.98786E-07	1.46696E-05	1.97577E-05
11	5.82409E-06	1.28781E-06	9.75087E-06	1.76409E-05
14	5.88926E-06	5.46349E-07	2.2569E-05	2.04841E-05
17	6.12633E-06	5.3589E-07	2.24037E-05	2.24936E-05
15	6.5536E-06	9.41038E-07	2.08611E-05	2.07385E-05
16	6.80348E-06	5.55878E-07	2.70394E-05	2.41253E-05
20	8.43128E-06	6.81506E-07	3.17415E-05	3.11744E-05
19	8.93445E-06	1.0596E-06	3.60981E-05	2.64718E-05
18	9.37042E-06	1.0525E-06	3.71991E-05	2.90604E-05
21	1.13043E-05	4.0083E-07	4.61499E-05	4.6921E-05
26	1.47051E-05	2.95773E-07	7.96294E-05	5.13739E-05
24	1.62214E-05	3.52823E-07	7.99385E-05	6.15102E-05
23	1.68243E-05	1.65319E-06	5.85783E-05	6.10681E-05
22	1.79551E-05	4.66959E-07	8.87535E-05	6.67871E-05
25	2.54283E-05	1.75346E-06	9.89428E-05	9.63112E-05
27	2.56217E-05	6.01196E-06	5.33016E-05	6.56561E-05
29	2.81352E-05	3.30777E-07	0.000182042	8.20919E-05
30	3.25633E-05	1.87855E-06	0.000165536	0.000103013
31	4.42185E-05	1.46049E-06	0.000235956	0.00014844
28	4.6869E-05	5.20202E-06	0.000154712	0.000167006

**Figure 3:**
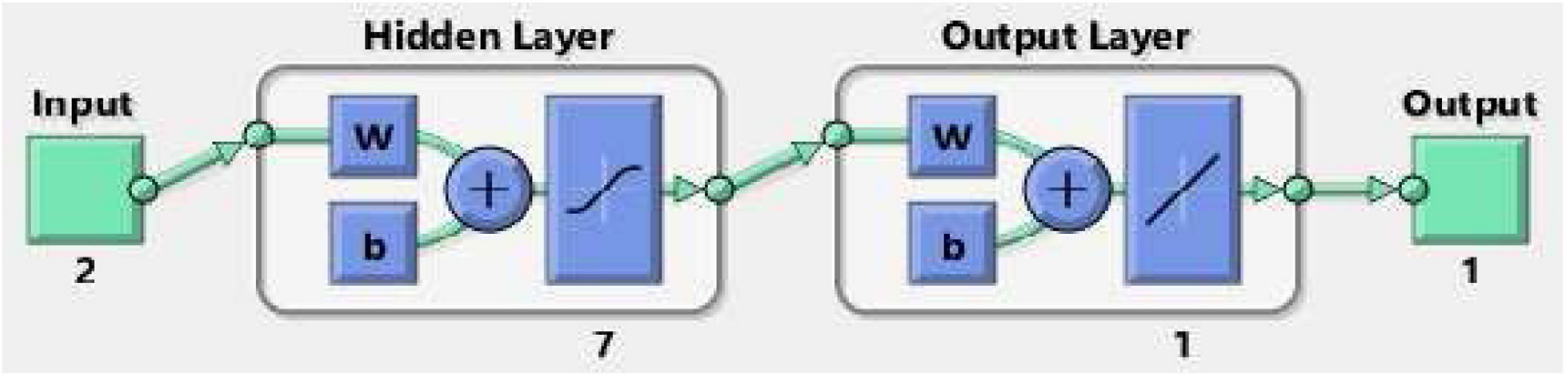
The architecture of ANN.

The architecture of the best ANN is shown in Figure 3.

In this ANN the tangent sigmoid is considered as the activation function and Levenberg Marquardt as the learning algorithm. In Table 2, the predicted data points by the ANN were presented. More than two-thirds of the data points are used for train and the rest of the data points are divided into two parts equally and used for validation and test. In Table 2, the blue, green, and red colors refer to the train points, validation, and test data points, respectively.

**Table 2:** The targets and ANN outputs.

Target	ANN Outputs
0.249	0.248869742
0.254	0.254049596
0.256	0.256006554
0.26	0.258436389
0.262	0.261904563
0.266	0.266180316
0.25	0.251367365
0.258	0.258279935
0.262	0.262426396
0.269	0.267093963
0.272	0.272648955
0.28	0.278838674
0.256	0.254184795
0.263	0.26279611
0.267	0.267978975
0.274	0.273196641
0.278	0.279115194
0.288	0.28462884
0.261	0.260977896
0.27	0.270117368
0.274	0.276391129
0.282	0.282339601
0.287	0.28698972
0.296	0.291452426
0.268	0.268195517
0.278	0.277162977
0.282	0.282185061
0.287	0.286665218
0.292	0.292353007
0.301	0.300497803
0.278	0.278258237
0.286	0.286089525
0.29	0.290810375
0.297	0.29624572
0.303	0.303163645
0.312	0.312625127
0.29	0.289731512
0.297	0.29724906
0.302	0.301925847
0.31	0.30700263
0.314	0.313967513
0.325	0.324786162

It can be seen that the predicted values are very close to the target data points. Another important criterion to judge the accuracy of results is the Correlation Coefficient. The correlation coefficient is defined as Eq. (1),

(1) $$\rho_{U,V}=\frac{E\left[\left(U-\mu_{U}\right)\left(V-\mu_{V}\right)\right]}{\sigma_{U}\sigma_{Y}}$$

In Eq. (1), U is the target and V is the predicted parameter, $\mu_{U}$ and $\mu_{V}$ are the mean values of U and V respectively. The standard deviations of U,V are $\sigma_{U},\sigma_{V}$ . Also ρ defines the correlation coefficient between the experimental and predicted values. In Table 3, the correlation coefficients for these neuron numbers are shown. In this table, the correlation coefficients also have been calculated for training, validation, test, and all data points.

**Table 3:** The correlation coefficients.

Neuron number	All correlation	Train correlation	Validation correlation	Test correlation
7	0.995841024	0.998173095	0.992745855	0.985776848
8	0.991557586	0.993946135	0.987198567	0.982331411
9	0.99125482	0.996788147	0.97726202	0.98289868
10	0.991332507	0.996505672	0.979866767	0.983689681
6	0.990323195	0.993546552	0.994402106	0.985228158
12	0.986131563	0.994615009	0.979715431	0.949196883
13	0.985305959	0.994180948	0.978782179	0.940809185
11	0.984291185	0.990926614	0.98062136	0.940798198
14	0.984719914	0.995705511	0.970811281	0.951357078
17	0.983368831	0.995725551	0.954561963	0.955944203
15	0.982064016	0.992779679	0.961334105	0.951394652
16	0.982157255	0.99554427	0.965851012	0.95147009
20	0.977269443	0.994975386	0.915534813	0.909114178
19	0.977044132	0.992361817	0.92979117	0.949634088
18	0.974667575	0.992102842	0.890259397	0.919773761
21	0.970968815	0.996922233	0.93438678	0.858038105
26	0.963273098	0.99758856	0.861394294	0.886830516
24	0.95948433	0.996887083	0.891636222	0.921035698
23	0.956408925	0.988209968	0.834790841	0.827560728
22	0.950213722	0.995996403	0.912140426	0.838429906
25	0.932747198	0.987596691	0.892840863	0.680383719
27	0.935725292	0.96079977	0.915316146	0.847093979
29	0.932411052	0.997447013	0.813070501	0.777120059
30	0.923746252	0.987745609	0.725091432	0.736761413
31	0.888390218	0.988788098	0.714433614	0.733361773
28	0.89077124	0.961063664	0.65718834	0.755554611

It can be seen that an ANN with 7 neurons in the hidden layer has a correlation coefficient very close to 1 and this proves that the outputs are compatible with experimental results.

### 3.2 Fitting method

The fitting method is another important method that is used for modeling the behavior of systems. In this work, a surface is fitted on the experimental data points in which, one axes are for temperature, one axes is for φ and the third axes shows k_nf_. To obtain an acceptable fitting result, combinatorial power functions were tested with different orders for temperature and φ and finally, the best one is selected (Eq. (2)), i.e., a first-order for temperature and the third order for φ,

(2) k(x,y) = p00 + p10∗x + p01∗y + p20∗xˆ2 + p11∗x∗y + p30∗xˆ3 + p21∗xˆ2∗y

In Eq. (2), x represents the φ and y represents the k_nf_ of nanofluid, and k(x,y) is the fitted result of k_nf_. The coefficients of this function are shown in Table 4

**Table 4:** The coefficients of the fitted surface.

Coefficients	Value
P00	0.2267
P10	0.0079
P01	0.0008362
P20	-0.008007
P11	0.000624
P30	0.00402
P21	-0.0001815

**Figure 4:**
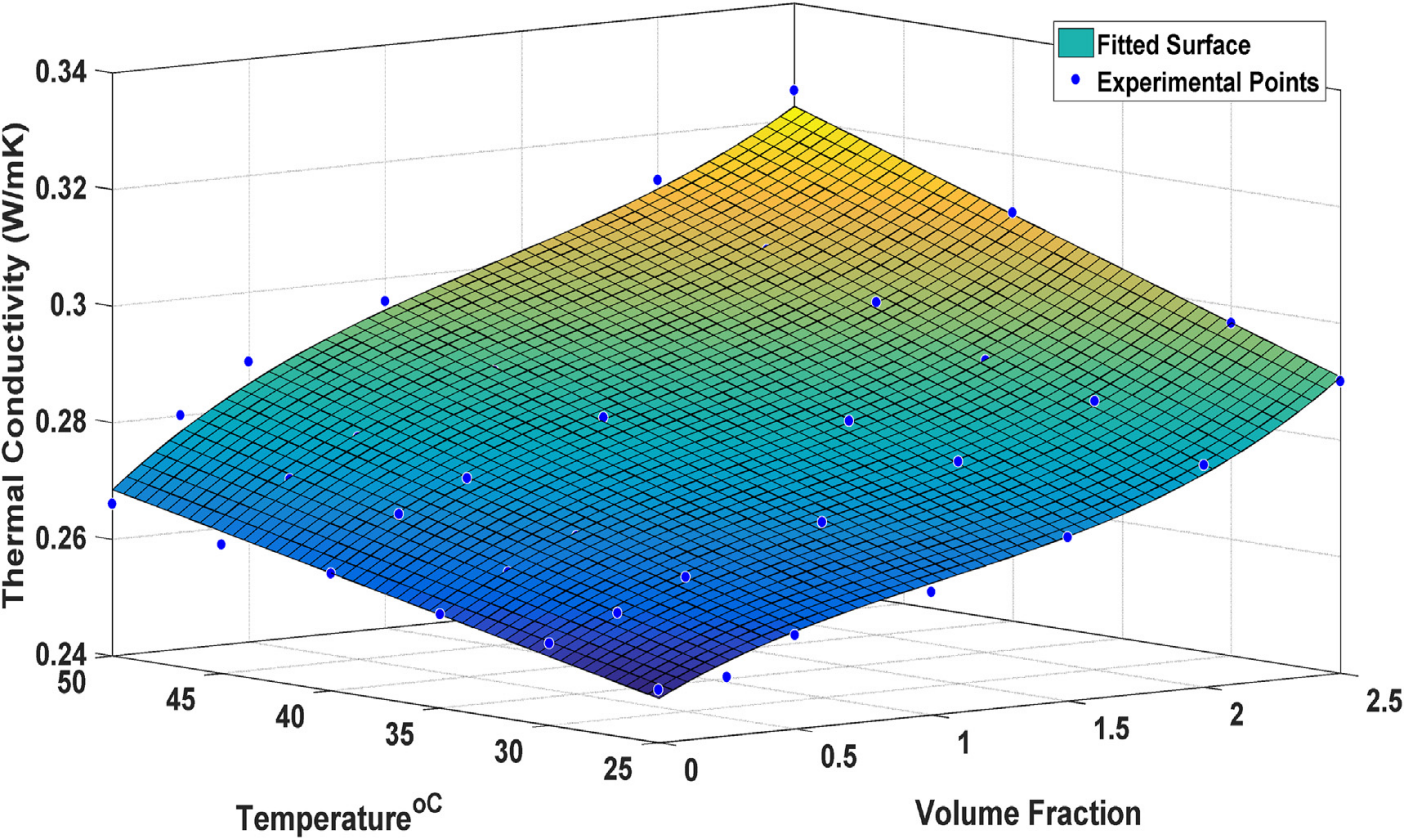
The fitted surface.

In Figure 4, the fitted surface is depicted. The dotted points are experimental data and the fitted surface is very close to these points. This surface depicts a better overview of the behavior of nanofluids. It can be seen that k_nf_ has a direct relationship to temperature and φ. The maximum k_nf_ occurs in the highest values of temperature and φ.

**Figure 5:**
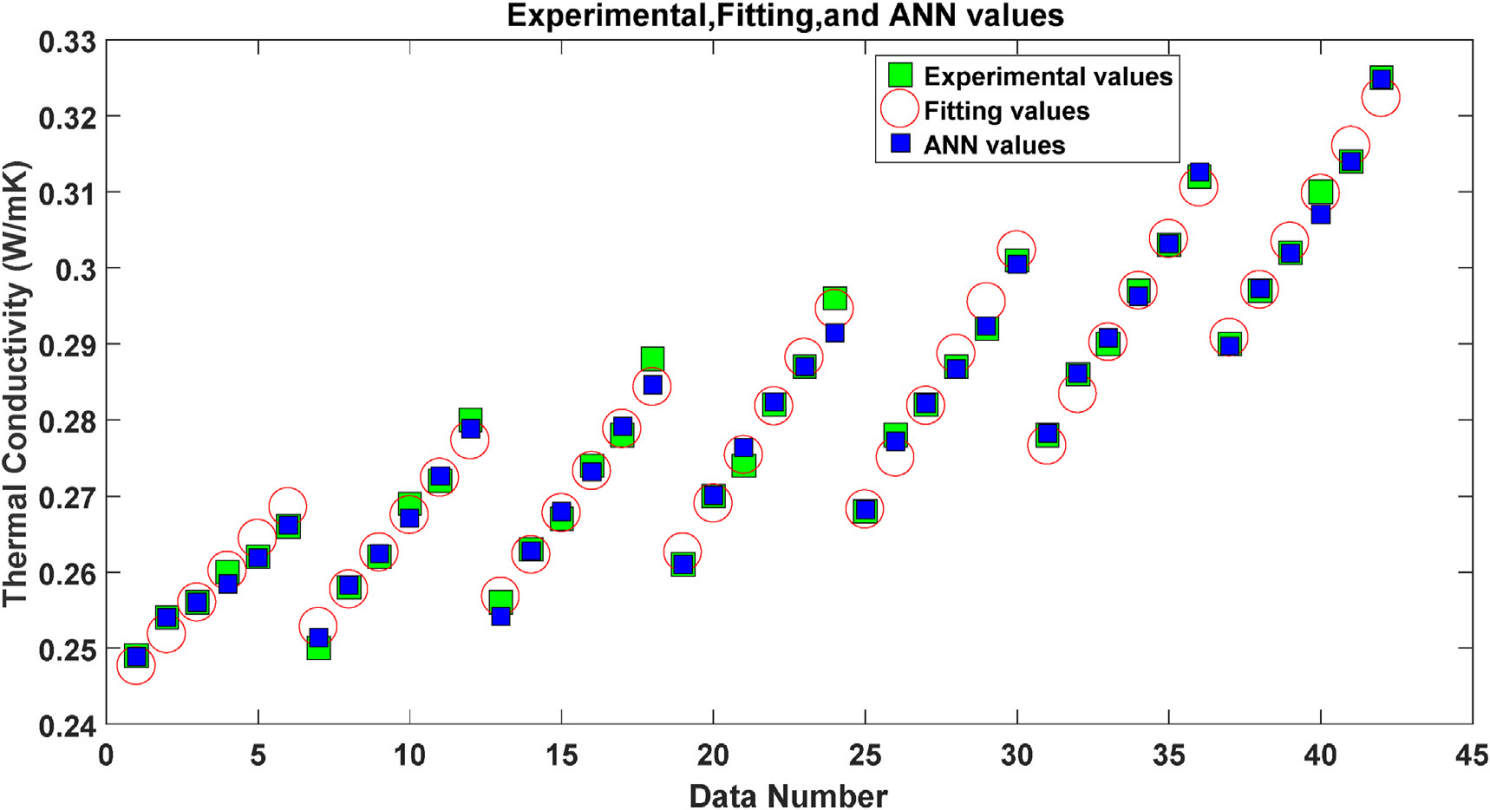
The Experimental data points, ANN and the Fitting results.

In Figure 5 the experimental data points, ANN outputs, and the fitted surface results are presented. It is found that both the ANN and fitting method have acceptable accuracy in predicting the k_nf_ based on the temperature and φ. It can be seen that ANN and fitting methods are near the experimental data points, and both these methods are accurate enough to predict k_nf_.

**Figure 6:**
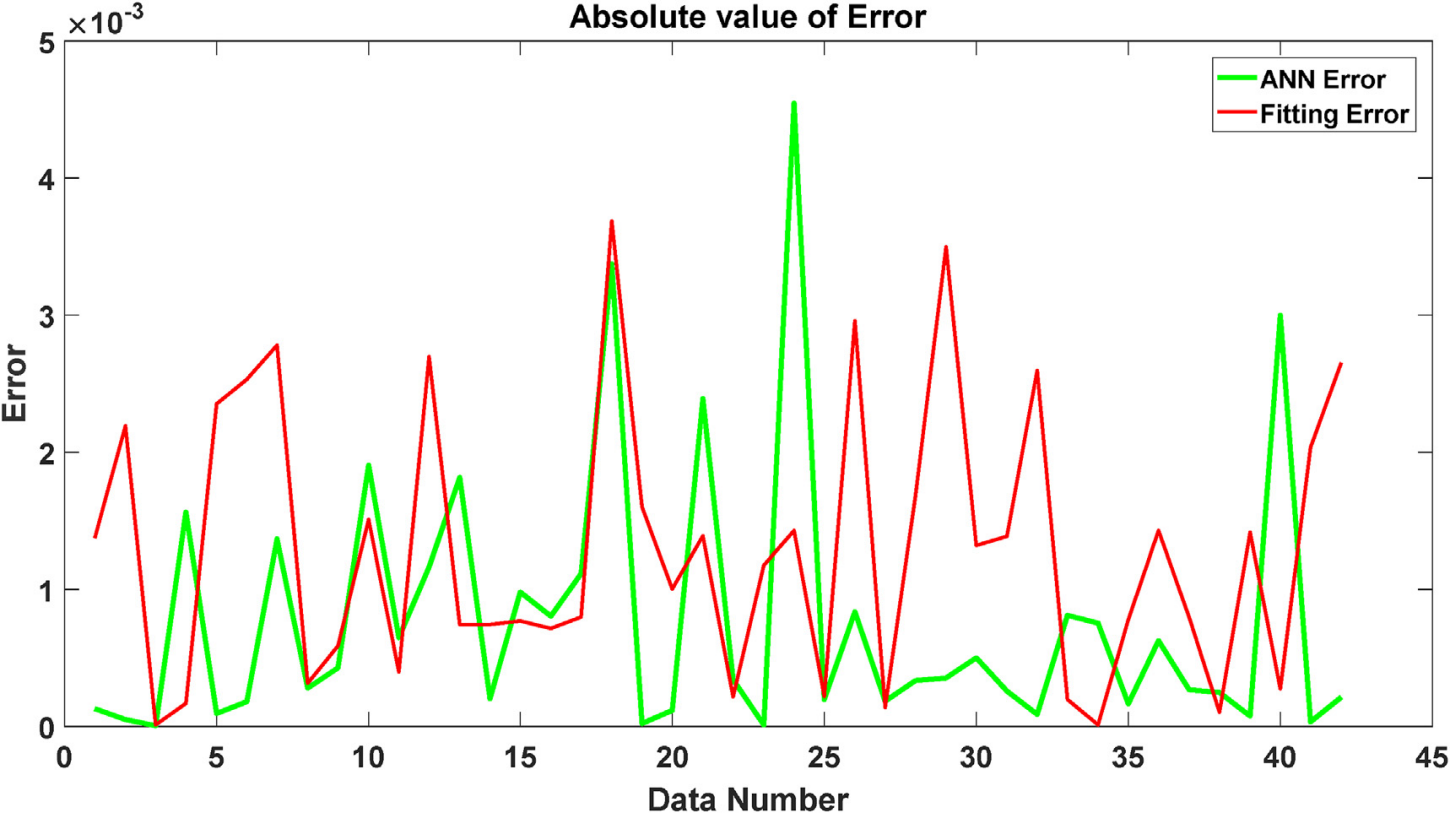
The error of ANN and fitting Method.

To have a better understanding of the results of these two methods, the errors of these methods are presented in Figure 6.

In Figure 4 the absolute values of errors are shown. It was assumed that the experimental data are our reference values; however, the error of extracting the experimental data may be more than 0.003, and we tried to predict these reference values. It can be seen that both methods have small errors but the ANN in most data points has smaller errors.

## 4 Conclusion

In this paper, the k_nf_ of cerium oxide/ethylene glycol nanofluid is predicted for different temperatures and φ by ANN and fitting methods. The important results of the research can be summarized as follows:

• An ANN with 7 neurons has a correlation coefficient very close to 1 and this proves that the outputs are compatible with experimental results.
• The ANN could predict the thermal behavior of this nanofluid more accurately.
• Both methods have small errors but the ANN in most data points has smaller errors.
• This method can decrease the lab costs and can obtain k_nf_ of this nanofluid for this range.

## Declarations

### Author contribution statement

Behrooz Ruhani, Mansour Taheri Andani, Azher M. Abed, Ghassan Fadhil Smaisim, Salema K. Hadrawi: Conceived and designed the experiments; Analyzed and interpreted the data; Contributed reagents, materials, analysis tools or data, Wrote the paper.

Nima Sina, Davood Toghraie: Conceived and designed the experiments; Performed the experiments; Analyzed and interpreted the data; Contributed reagents, materials, analysis tools or data.

### Funding statement

This research did not receive any specific grant from funding agencies in the public, commercial, or not-for-profit sectors.

### Data availability statement

No data was used for the research described in the article.

### Declaration of interest’s statement

The authors declare no conflict of interest.

### Additional information

No additional information is available for this paper.
